# Functionality, complications, and survivorship of total shoulder arthroplasty in patients under 60 years old

**DOI:** 10.1016/j.jor.2024.04.007

**Published:** 2024-04-11

**Authors:** Louis W. Barry, Erryk S. Katayama, John S. Barnett, Brent L. Henderson, Akshar V. Patel, Gregory L. Cvetanovich, Julie Y. Bishop, Ryan C. Rauck

**Affiliations:** Department of Orthopaedics, The Ohio State University College of Medicine, Columbus, OH, USA

**Keywords:** Total shoulder arthroplasty, Young patients, Complications, Survivorship

## Abstract

**Background:**

As total shoulder arthroplasty (TSA) expands to younger patients, it is crucial to weigh the benefits of early intervention against potential complications and implant longevity in patients under 60 years of age. This study examines mid-term outcomes in this patient subset.

**Methods:**

Between 2009 and 2019, a retrospective analysis was conducted on 50 patients (25 male, 25 female) who underwent anatomic TSA (TSA) under the age of 60 with minimum 5 years follow-up. Demographic and baseline variables were extracted from medical records. Pre-operative and post-operative outcomes of range of motion (ROM) and strength were recorded. Patient-reported outcomes (PROs) were obtained.

**Results:**

Fifty patients were followed for an average of 8.7 ± 2.4 years, having a mean age of 54.1 ± 8.4 years. Comparison of pre-operative and post-operative measurements revealed significant improvements in active ROM, including external rotation (ER) (p < 0.0001), forward elevation (FE) (p < 0.0001), and internal rotation (IR) (p = 0.0001). There were significant improvements in functional strength scores, including ER (p = 0.0005) and FE (p = 0.0002). PROs included visual analog scale (VAS) (2.2 ± 2.6), Single Assessment Numeric Evaluation (SANE) (80.3 ± 17.6), American Shoulder and Elbow Surgeons (ASES) score (76.4 ± 22.8), and Simple Shoulder Test (SST) (8.9 ± 3.2). The 5-year and 10-year implant survival rates were found to be 98.0 % and 83.3 %, respectively. There were 7 postoperative complications in 5 patients (14.0 %), including glenoid loosening (n = 2), infection (n = 1), atraumatic instability (n = 1), lesser tuberosity avulsion (n = 1), painful arthroplasty (n = 1) and traumatic rotator cuff insufficiency (n = 1). Subsequently, all 5 patients underwent revision shoulder arthroplasty at an average of 6.5 years after the initial procedure.

**Conclusion:**

Positive mid to long-term outcomes, including significant improvements in ROM and strength, along with high 5-year and 10-year implant survival rates support TSA as an effective treatment option for patients under the age of 60.

## 1 Introduction

Anatomic total shoulder arthroplasty (TSA) is a commonly utilized treatment option in painful and advanced degenerative conditions of the shoulder.^1^ While the procedure has demonstrated consistent success in elderly populations, characterized by enhanced range of motion (ROM) and reduced pain with rare occurrences of revision surgery, its application in patients under 60 years of age presents unique challenges. Younger individuals often present with more complex pathology and preoperative disability, along with heightened postoperative expectations due to increased demand for arm usage and extended replacement lifespan.^2^ Thus, it is especially important to understand the mid-long term outcomes younger populations face following TSA.

The primary concern for TSA in the younger demographic lies in implant use and longevity – particularly glenoid loosening.^3^ Despite existing alternatives like debridement, glenoid resurfacing, and hemiarthroplasty, TSA has shown more consistent and positive outcomes.^4^^,^^5^ Current research in this field is limited, with available studies focusing largely on short to mid-term outcomes for patients under the age of 65. Recognizing the significance of mid to long-term outcomes for younger patients, this study aims to analyze postoperative outcomes and complications in patients under 60 years of age with minimum 5 years follow-up. We hypothesize that patients under the age of 60 will experience long-term benefits from anatomic TSA, with minimal postoperative complications.

## 2 Materials and Methods

### 2.1 Patient population

A study investigating shoulder functionality outcomes and implant survival among patients under 60 years of age was conducted retrospectively using a case-control design. By analyzing institutional records that included the Current Procedural Terminology code 23472, individuals identified had undergone their primary anatomic TSA procedure between 2009 and 2020, were at or under 60 years of age, and had minimum follow-up of 5 years. Review of patient medical records involved collecting demographic details, comorbidities, and lifestyle factors (Charlson Comorbidity Index, preoperative American Society of Anesthesiologist physical status classification score, and body mass index). Additionally, clinical data including the indication of surgery, arthroplasty type, complications, follow-up duration, and physical exam measurements before and after arthroplasty, such as ROM and strength in FE, ER, and IR, was obtained. Implant survival was defined as no revision surgery after their primary shoulder arthroplasty. Exclusion criteria included patients with inaccessible records from outside institutions, clinical follow-up of less than 5 years, and age of over 60 years old at time of shoulder arthroplasty.

### 2.2 Operative technique

This study was conducted at a single institution and involved procedures performed by seven different surgeons. All primary operations used an anatomic TSA approach. The primary implant utilized was the Zimmer Trabecular Metal Press-Fit system, with surgical approaches involving either lesser tuberosity osteotomy or tenotomy for subscapularis management. Following the surgical procedure, a comprehensive post-operative rehabilitation program was enacted. It involves 6 weeks of sling immobilization, with passive ROM physical therapy introduced during the third week. As patients progressed, physical therapy evolved to encompass both supported active ROM and active ROM exercises. The rehabilitation regimen culminated with strength-building exercises aimed at achieving complete recovery and enabling a return to normal activities. As part of their post-operative care, patients were advised to abstain from engaging in adduction, IR, and cross body movements for a minimum of 3 months.

### 2.3 Clinical evaluation

In this study, we evaluated the effectiveness of the surgical interventions by examining all patients at several clinical endpoints. These endpoints, including ROM and strength scores in FE, ER, and IR, were recorded at last clinical follow-up by each surgeon and retrieved through retrospective review. IR was categorized based on the criteria established by Amroodi et al. ^6^ and ROM scores were recorded both preoperatively and during the final postoperative follow-up appointment. Additionally, PROs were obtained using VAS, SANE, ASES, and SST scores. To gather comprehensive PROs and information on any surgical revisions not documented in electronic medical records, we conducted a thorough phone questionnaire with patients in June and July 2023.

### 2.4 Statistical analysis

The study employed several statistical methods to analyze the data. For comparing continuous variables, the student's t-test was used, and paired when comparing preoperative and postoperative measurements. Trends and comparisons among ordinal variables were assessed using the Wilcoxon signed-rank test. We determined statistical significance (p < 0.05), and all analyses were performed using two-sided tests. We constructed Kaplan-Meier survival curves with a 95 % confidence interval to evaluate implant survival. The presentation of results was in the form of mean ± standard deviation. All analyses were carried out using Stata/SE 17.0 (StataCorp, College Station, TX).

## 3 Results

### 3.1 Study population

During the study period, we identified 50 eligible patients as receiving primary anatomic TSA under the age of 60 while having a minimum of 5 years follow-up. The average age at time of surgery was 54.1 ± 5.6 years (range 39.1–59.9 years). The mean duration of postoperative follow-up time was 8.7 ± 2.4 years. An equal distribution of male and female patients was observed, with 25 male and 25 female shoulders included. The average body mass index (BMI) was 32.4 ± 8.4 kg/m^2^, CCI was 1.5 ± 1.2, and ASA score was 2.4 ± 0.6. The indications for TSA included osteoarthritis (n = 46), avascular necrosis (n = 2), rheumatoid arthritis (n = 1), and proximal humerus osteosarcoma (n = 1).

### 3.2 Clinical outcomes

Patients aged less than 60 years old undergoing primary TSA demonstrated significant improvements in ROM and strength postoperatively. FE increased from 114° ± 29° preoperatively to 147° ± 23° postoperatively (p < 0.0001), ER increased from 33° ± 16° preoperatively to 46° ± 11° postoperatively (p < 0.0001), and IR improved from sacrum preoperatively to L3 postoperatively (p = 0.0001). During strength assessment, patients showed statistically significant enhancements in their FE, progressing from a preoperative score of 4+/5 to a postoperative score of 5/5 (p = 0.0005). Similarly, ER improved from 4+/5 preoperatively to 5/5 postoperatively (p = 0.0002) (Table 1). Postoperatively, patients reported ASES scores of 76.4 ± 22.8, SST scores of 8.9 ± 3.2, VAS scores of 2.2 ± 2.6, and SANE scores of 80.3 ± 17.6 (Table 2).

**Table 1:** Preoperative vs Postoperative Outcomes.

	ER ROM	FE ROM	IR ROM	ER Strength	FE Strength	IR Strength
**Preoperative**	33 ± 16	114 ± 30	Sacrum	4+/5	4+/5	5/5
**Postoperative**	46 ± 11	147 ± 23	L3	5/5	5/5	5/5
**P value**	<0.0001	<0.0001	0.0001	0.0005	0.0002	0.0537

Note: ROM, range of motion; ER, external rotation; FE, forward elevation; IR, internal rotation.

**Table 2:** Patient reported outcomes.

Measure	Mean ± SD	Range
**VAS**	2.2 ± 2.6	0–10
**SANE**	80.3 ± 17.6	5–100
**ASES**	76.4 ± 22.8	8.3–1000
**SST**	8.9 ± 3.2	0–12

Note: SD, standard deviation; VAS, visual analog scale; SANE, Single Assessment Numeric Evaluation; ASES, American Shoulder and Elbow Surgeons score; SST, Simple Shoulder Test.

### 3.3 Complications/revisions

**Fig. 1:**
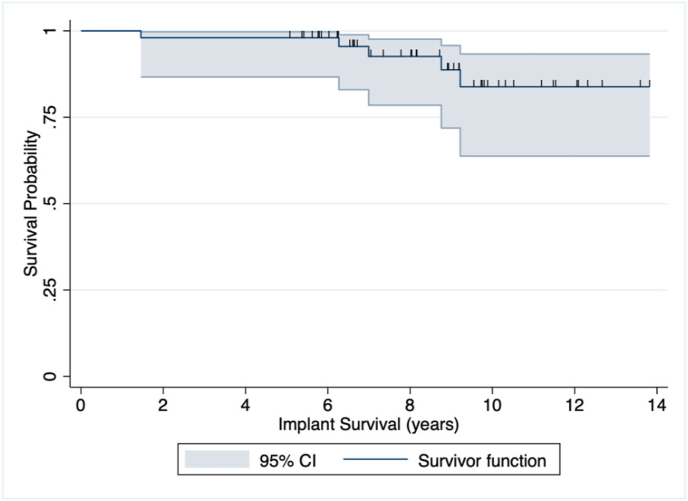
Implant survival.

A total of 7 complications occurred in 5 patients (14 %). Of these patients, 2 experienced glenoid loosening. One patient underwent arthroscopic removal of the glenoid component 6.3 years after the primary TSA and subsequently was converted to reverse TSA 7.3 months later due to painful arthroplasty. The second patient with glenoid loosening underwent revision TSA 9.2 years after the primary TSA. Another patient required revision TSA for atraumatic instability 7.0 years postoperatively. Two patients underwent revision to reverse TSA, one for traumatic rotator cuff insufficiency 1.5 years postoperatively and the other with multiple revisions. The latter initially underwent revision to TSA for lesser tuberosity avulsion, followed by an infection 6.5 months later, leading to revision to reverse TSA (Table 3). The rate of implant survival was 98.0 % at 2-years, 98.0 % at 5-years, and 83.8 % at 10-years (Fig. 1).

**Table 3:** Patient demographics, complications, and revisions.

	Age (yr)	Sex	Shoulder	Indication	Follow-up (yr)	Complication	Revision
**Patient 1**	41.9	Male	Right	Osteoarthritis	5.0	–	–
**Patient 2**	55.5	Female	Right	Osteoarthritis	5.2	Traumatic rotator cuff insufficiency	RTSA
**Patient 3**	56.4	Male	Right	Osteoarthritis	5.3	–	–
**Patient 4**	42.5	Female	Right	Osteoarthritis	5.4	–	–
**Patient 5**	55.1	Female	Left	Osteoarthritis	5.6	–	–
**Patient 6**	58.8	Male	Left	Osteoarthritis	5.7	–	–
**Patient 7**	42.1	Male	Right	Proximal humerus osteosarcoma	5.8	–	–
**Patient 8**	58.7	Male	Left	Osteoarthritis	5.8	–	–
**Patient 9**	46.3	Male	Right	Osteoarthritis	6.0	–	–
**Patient 10**	58.3	Male	Right	Osteoarthritis	6.2	–	–
**Patient 11**	57.0	Male	Left	Osteoarthritis	6.2	–	–
**Patient 12**	40.7	Female	Left	Osteoarthritis	6.5	–	–
**Patient 13**	39.1	Female	Right	Osteoarthritis	6.6	Atraumatic instability	ATSA
**Patient 14**	57.1	Male	Left	Osteoarthritis	6.6	–	–
**Patient 15**	59.0	Female	Right	Avascular necrosis	6.6	–	–
**Patient 16**	47.5	Male	Right	Osteoarthritis	6.6	–	–
**Patient 17**	58.0	Female	Left	Osteoarthritis	6.7	–	–
**Patient 18**	56.8	Female	Left	Osteoarthritis	7.0	–	–
**Patient 19**	59.1	Female	Left	Osteoarthritis	7.3	–	–
**Patient 20**	58.5	Male	Left	Osteoarthritis	7.7	–	–
**Patient 21**	58.2	Male	Left	Osteoarthritis	8.0	–	–
**Patient 22**	59.9	Female	Left	Osteoarthritis	8.0	–	–
**Patient 23**	56.1	Female	Right	Osteoarthritis	8.1	–	–
**Patient 24**	56.8	Male	Right	Osteoarthritis	8.1	–	–
**Patient 25**	55.0	Male	Right	Osteoarthritis	8.7	–	–
**Patient 26**	52.2	Female	Right	Rheumatoid Arthritis	8.9	–	–
**Patient 27**	56.6	Female	Left	Avascular Necrosis	8.9	–	–
**Patient 28**	55.7	Female	Left	Osteoarthritis	9.0	–	–
**Patient 29**	58.0	Female	Right	Osteoarthritis	9.2	–	–
**Patient 30**	50.2	Male	Left	Osteoarthritis	9.2	–	–
**Patient 31**	56.5	Male	Right	Osteoarthritis	9.5	–	–
**Patient 32**	49.2	Female	Left	Osteoarthritis	9.7	–	–
**Patient 33**	53.6	Male	Right	Osteoarthritis	9.7	–	–
**Patient 34**	59.1	Female	Right	Osteoarthritis	9.8	–	–
**Patient 35**	58.5	Male	Left	Osteoarthritis	9.9	–	–
**Patient 36**	55.8	Male	Left	Osteoarthritis	10.1	–	–
**Patient 37**	56.7	Female	Left	Osteoarthritis	10.3	–	–
**Patient 38**	59.6	Female	Right	Osteoarthritis	10.5	–	–
**Patient 39**	54.2	Female	Right	Osteoarthritis	10.9	Glenoid loosening and painful arthroplasty	HA and ATSA
**Patient 40**	59.5	Female	Left	Osteoarthritis	10.9	Glenoid loosening	ATSA
**Patient 41**	54.6	Female	Left	Osteoarthritis	11.2	–	–
**Patient 42**	56.5	Male	Left	Osteoarthritis	11.5	–	–
**Patient 43**	48.1	Female	Right	Osteoarthritis	11.5	–	–
**Patient 44**	51.7	Male	Left	Osteoarthritis	12.0	–	–
**Patient 45**	55.1	Male	Left	Osteoarthritis	12.1	–	–
**Patient 46**	44.3	Male	Right	Osteoarthritis	12.1	Traumatic lesser tuberosity avulsion and infection	ATSA and RTSA
**Patient 47**	57.6	Female	Right	Osteoarthritis	12.3	–	–
**Patient 48**	53.7	Female	Left	Osteoarthritis	12.6	–	–
**Patient 49**	58.3	Male	Right	Osteoarthritis	13.6	–	–
**Patient 50**	54.8	Male	Left	Osteoarthritis	13.8	–	–
**Mean ± SD or n**	54.1 ± 5.6	25 male and 25 female	24 right and 26 left		8.7 ± 2.4		

Note: ATSA, anatomic total shoulder arthroplasty; RTSA, reverse total shoulder arthroplasty; HA, hemiarthroplasty; SD, standard deviation.

## 4 Discussion

The literature on primary TSA in younger patients presents conflicting conclusions, with some studies suggesting unsatisfactory outcomes. For instance, in a long-term study on 36 TSA patients aged under 50 years, Schoch et al. ^7^ reported 11 unsatisfactory outcomes due to revision (n = 6) and limited motion (n = 5), suggesting caution on performing TSA in younger patients. In a systematic review of 6 studies on primary TSA in patients under 65, Roberson et al. ^2^ found inferior postoperative SST and ASES scores in young patients when compared to the broader TSA population, emphasizing hesitancy on TSA in younger individuals. Similarly, a study of 44 TSA patients under 50 concluded that although postoperative pain and function are improved, subjective patient satisfaction may not correlate with those benefits.^8^ Roberson et al. ^2^ also contended that the appeal of TSA in the younger population stems from the absence of more advantageous alternatives. Despite arguments endorsing conservative management, such as arthroscopic debridement, evidence supporting its long-term benefits compared to TSA is lacking.^4^^,^^5^.

There is a paucity in mid and long-term outcomes in the literature on TSA among younger patients. We observed significant improvements in ROM, strength, and patient-reported outcomes in TSA patients under 60 with a minimum follow-up of 5 years. These findings align with recent retrospective studies among similar cohorts, like Bronchin et al. ^9^, suggesting positive outcomes. However, concerns for TSA in the younger patient persist, primarily lying within the variability in reported implant survival rates, postoperative complications, and subsequent revisions.^9^^,^^2^.

Implant survivorship in younger patients undergoing TSA have been found to be as low as 89 % at 5 years, 63 % at 10 years, and 60 % at 20 years.^10^^,^^8^^,^^11^ Yet, a systematic review by Davies et al. ^12^ reported a 82.3 % implant survival rate at 10 years for TSA patients under 65. Our study found a survival rate of 98.0 % and 83.8 % at 5 years and 10 years, respectively, reflecting a relatively high implant survivorship despite a younger patient population. It is easy to understand the concern of TSA in younger patients regarding implant survivorship, as implant survival is expected to decrease over time. The growing occurrence of TSA in individuals under the age of 65, coupled with the rising life expectancy in the US, will result in a larger population of patients who underwent TSA at a young age. ^13^ Notably, Bronchin et al. ^9^ and Deshmukh et al. ^14^ propose promising 20-year survivorship rates of 80.1 % and 85.0 %, respectively, for patients under 60. Nevertheless, the varying rates of implant survival rates across the literature suggests a difficulty predicting TSA survival in younger adults.

Postoperative complications are often a significant concern in younger patients undergoing TSA. The most common postoperative complication with subsequent revision in patients undergoing TSA is glenoid loosening, accounting for 24 % of all complications. ^15^ Roberson et al. 2 reported rates of overall postoperative complications ranging from 4.2 % to 15.2 % in TSA patients under 65, with glenoid loosening being the most common complication and reason for revision (53 %). Our study observed a complication rate of 14 %, with 7 complications occurring in 5 patients and all patients undergoing revision. Of these revisions, 2 of 5 were due to glenoid loosening (40 %). Other complications leading to revision included atraumatic instability, traumatic rotator cuff insufficiency, and lesser tuberosity avulsion. It is possible to attribute these complications occurring in younger patients to a more active lifestyle, with patients under 60 being more active in sports, work, or otherwise higher risk activities. ^10^^,^^16^ Furthermore, one factor that may account for the variability in age-related postoperative complications may be linked to the presence of more complex pathologies in younger patients.^17^ In a study of 1404 shoulders in patients under 60 years old undergoing primary shoulder arthroplasty, there was a resulting 2.35 % (n = 55) rate of periprosthetic joint infection.^18^ Our study, with no postoperative infections following primary TSA but a 2.0 % (n = 1) infection rate following revision TSA, suggests that a more complex pathology, including multiple revisions, may contribute to postoperative complications.

Our cohort had an average obese BMI of 32.4 ± 8.4 kg/m^2^, which may be a predictor for earlier intervention or account for differences in outcomes. One retrospective study found that patients with a higher BMI required TSA at a younger age when comparing 34 obese and 94 non-obese patients undergoing primary TSA.^19^ In a recent study by Katayama et al., 113 obese TSA patients were found to have no differences in post-operative functional measurements, implant survival, or complications compared to 317 non-obese TSA patients.^20^ Yet, they did not find BMI as a predictor of earlier intervention for anatomic TSA. Furthermore, Reid et al. concluded that both obese (n = 635) and non-obese (n = 885) patients experience significant improvements in ROM, pain, and other functional outcome scores following TSA.^21^ It is difficult to draw a firm conclusion of the effect on BMI on outcomes, as the current literature on this topic is mixed.^22^.

Our study has several notable limitations. We utilized a retrospective case-control design, which inherently has limitations in terms of data collection and potential biases. By relying on existing records, the quality and completeness of those records may vary, affecting the accuracy and reliability of the findings. Additionally, this research took place at a single institution, involving procedures performed by seven different surgeons. This may introduce variability in surgical techniques, postoperative care, and rehabilitation protocols, potentially impacting the consistency of outcomes. The relatively small sample size of 50 patients in this study could limit the generalizability of the findings and influence the statistical power. Moreover, there is potential bias in patient reported outcomes, as patients with unsatisfactory outcomes may have been unwilling to participate in the questionnaire.

## 5 Conclusion

Patients under the age of 60 years can have successful outcomes with anatomic TSA. Survivorship remains excellent at 5 years and 10 years, with clinical outcomes demonstrating significant improvements in functional markers as well as PROs. Future directions may include comparative long-term outcomes between patients under and over 60 years old in an attempt to establish non-inferiority of outcome for patient populations. While unique patient factors are paramount in surgical recommendations, our work suggests that TSA in patients under 60 years old is both safe and effective.

## Disclaimer

None.

## Funding/sponsorship

None.

## Ethical statement

This study was approved by the Biomedical Institutional Review Board of The Ohio State University.

## Guardian/patient's consent

Consent was not required by our institution for this retrospective study.

## CRediT authorship contribution statement

**Louis W. Barry:** Project administration, Data curation, Visualization, Writing – original draft. **Erryk S. Katayama:** Data curation, Writing – original draft. **John S. Barnett:** Data curation, Writing – original draft. **Brent L. Henderson:** Formal analysis, Visualization, Writing – original draft. **Akshar V. Patel:** Conceptualization, Project administration, Writing – review & editing. **Gregory L. Cvetanovich:** Conceptualization, Writing – review & editing. **Julie Y. Bishop:** Conceptualization, Writing – review & editing. **Ryan C. Rauck:** Conceptualization, Supervision, Writing – review & editing.

## Declaration of competing interest

None.
